# Non-coding RNA Contribution to Thoracic and Abdominal Aortic Aneurysm Disease Development and Progression

**DOI:** 10.3389/fphys.2017.00429

**Published:** 2017-06-16

**Authors:** Yuhuang Li, Lars Maegdefessel

**Affiliations:** ^1^Vascular Biology Unit, Department of Vascular and Endovascular Surgery, Klinikum rechts der Isar der Technical University of MunichMunich, Germany; ^2^Department of Medicine, Karolinska InstitutetStockholm, Sweden

**Keywords:** aortic aneurysm, non-coding RNA (ncRNA), MicroRNA (miRNA), gene expression regulation, vascular diseases, long non-coding RNA (lncRNA)

## Abstract

Multiple research groups have started to uncover the complex genetic and epigenetic machinery necessary to maintain cardiovascular homeostasis. In particular, the key contribution of non-coding RNAs (ncRNAs) in regulating gene expression has recently received great attention. Aneurysms in varying locations of the aorta are defined as permanent dilations, predisposing to the fatal consequence of rupture. The characteristic pathology of an aneurysm is characterized by progressive vessel wall dilation, promoted by dying vascular smooth muscle cells and limited proliferation, as well as impaired synthesis and degradation of extracellular matrix components, which at least partially is the result of transmural inflammation and its disruptive effect on vessel wall homeostasis. Currently no conservative pharmacological approach exists that could slow down aneurysm progression and protect from the risk of acute rupture. In the recent past, several non-coding RNAs (mainly microRNAs) have been discovered as being involved in aneurysm progression throughout varying locations of the aorta. Exploring ncRNAs as key regulators and potential therapeutic targets by using antisense oligonucleotide strategies could open up promising opportunities for patients in the near future. Purpose of this current review is to summarize current findings and novel concepts of perspectivly utilizing ncRNAs for future therapeutic and biomarker applications.

## Introduction

Aortic aneurysms (AAs) are asymmetrical dilations of the aorta with diameters >1.5 times the normal size (Kent, 2014). They are most commonly located in the infrarenal abdominal aorta but can also be found in the thoracic aorta. The overall prevalence of abdominal AAs (AAAs) is 6% in men and 1.6% in women (Li et al., 2013); the incidence for thoracic AAs (TAAs) is roughly 10/10,000 in our population (Elefteriades et al., 2015). Fatal outcomes due to aortic rupture occur when intraluminal pressures exceed the capacity of the arterial wall, with mortality rates as high as 80% (Golledge and Norman, 2011). It is estimated that aneurysm ruptures account for nearly 15,000 deaths in the United States annually (Lloyd-Jones et al., 2009). Most of these fatalities are due to abdominal aneurysms, with thoracic and thoracoabdominal aneurysms accounting for 1 to 4% of all aneurysm-related deaths (Lindsay and Dietz, 2011). The asymptomatic characteristic of aneurysms makes their assessment challenging. Unless AAs rapidly increase or acutely rupture, or thrombi embolize into the distal arterial system, AAs behave as a silent disease (Kent, 2014).

Currently, the only available treatment of AAs is surgical intervention, such as prosthetic replacement (open surgery) or strengthening (endoprosthesis) of the aorta (Golledge et al., 2006). The latter endovascular repair has become the preferable option in the past decade, accounting for ~70% of AA procedures performed in Europe and North America (Schanzer et al., 2017; Williams and Brooke, 2017). However, clinically surgical intervention is only recommended when aneurysms are prone to rupture, since both interventional procedures carry surgical risks (Kent, 2014). Unfortunately, no effective pharmacological approaches have been approved to limit the progression or risk of aneurysm rupture in humans (Golledge et al., 2006). Obtaining a better understanding of the cellular mechanisms driving aneurysm development and progression is not only essential to fill this gap but is also important to identify new biomarkers and therapeutic targets.

**Figure 1:**
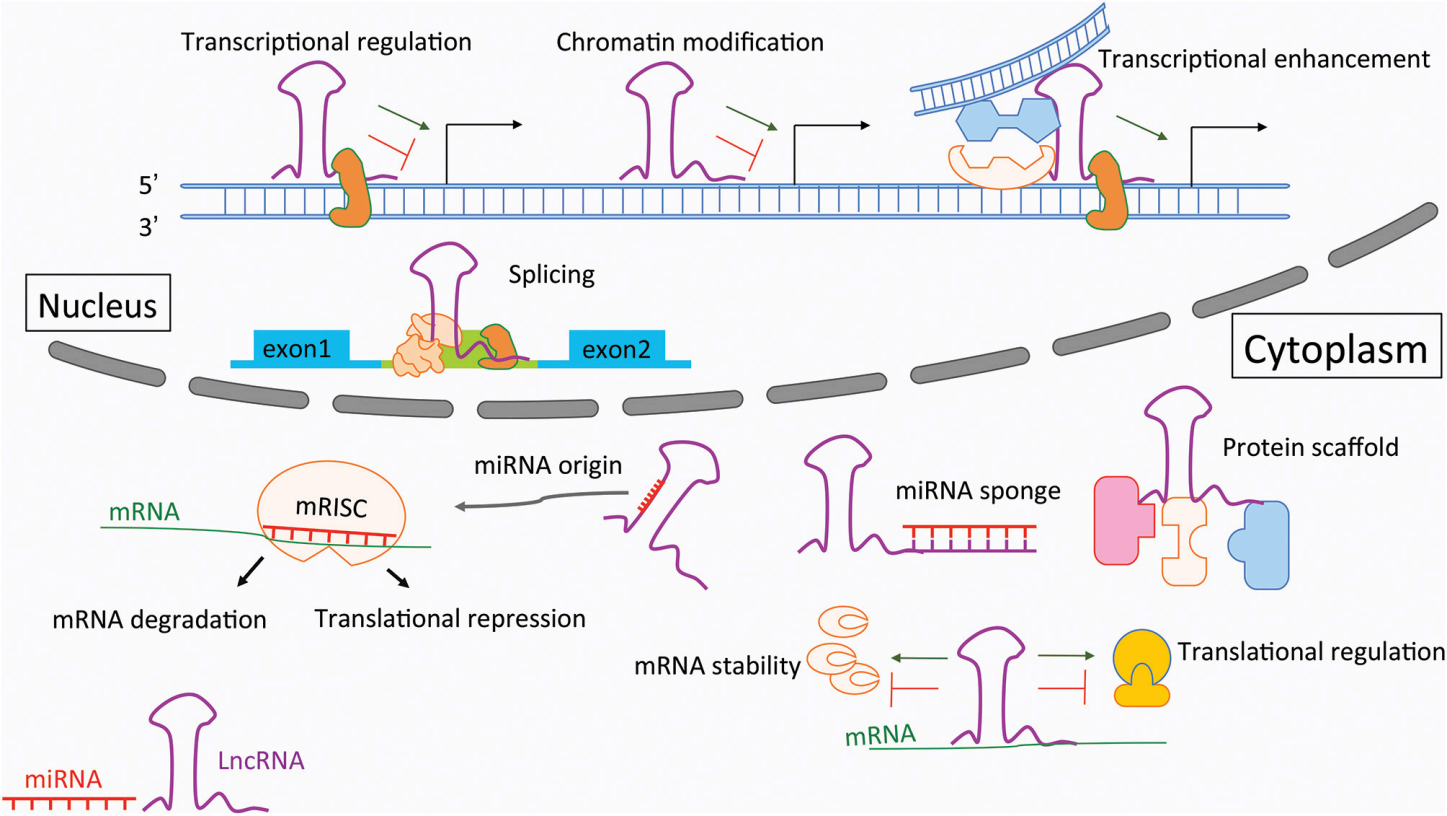
Mechanisms of action for non-coding RNAs during disease development and progression. Long non-coding RNAs (lncRNAs) regulate DNA processing via transcriptional regulation, chromatin modification, trsncriptional enhancement, as well as by affecting splicing mechanisms in the nucleus. Within the cytoplasm lncRNAs are involved by sponging microRNAs (miRNAs) or by sesrving as miRNA host transcripts (miRNA origin), as well as via playing an important role as protein scaffolds and affectors of mRNA stability and transcription. Mature, single-stranded miRNAs get processed into the messenger RNA induced silencing complex (mRISC) where they bind to messenger RNA (mRNA), augmenting gene expression either via translational repression, or mRNA degradation.

This review focuses on the contribution of non-coding RNAs (ncRNAs) to AA development and progression, and discusses their potential therapeutic value in this context. With the completion of human genome project in 2003, the “protein-centered” dogma of molecular biology was challenged with the discovery that while >98% of our genome is “non-coding,” it is still transcribed (Esteller, 2011). Accumulating evidence has shown that this refined orechstrating system of ncRNA mediators are powerful regulators of various aspects of cellular function and disease progression (Vidigal and Ventura, 2015; Engreitz et al., 2016; Figure 1). In general, ncRNAs are classified, based on an arbitrary cut-off size of 200 nucleotides (nt), into separate groups of small ncRNAs and long non-coding RNAs (lncRNAs). MicroRNAs (miRNAs) are the most well-studied group of small ncRNAs, and ~22 nt in length, whereas lncRNAs are larger than 200 nt and their distinct functions being largely unexplored (Cech and Steitz, 2014).

## Cellular mechanisms behind aortic aneurysm development and progression

The lack of pharmacological approaches to limit aneurysm progression and rupture mandates a better understanding of the pathogenesis and cellular mechanisms of aneurysm development. New mechanistic insights may identify potential therapeutic targets. Here, we summarize the pathologic mechanisms and related molecular characteristics behind thoracic and abdominal aneurysm development and progression.

## Thoracic aortic aneurysm

Despite the common phenotypic manifestations with AAAs (discussed below), TAAs exhibit a strong heritable pattern, with different pathophysiologies, risk factors, and evolutionary progression. For example, the known risk factors most associated with AAAs include cigarette smoking, increasing age, hypertension, hyperlipidemia, and atherosclerosis, whereas TAAs are primarily associated with connective tissue diseases, bicuspid aortic valve disease, and familial thoracic aneurysm syndrome. Nevertheless, perturbed extracellular matrix (ECM) homeostasis, transforming growth factor-β (TGF-β) signaling, and vascular smooth muscle cell (SMC) plasticity and survival have been proposed as important processes in TAA pathogenesis (Verstraeten et al., 2017). Recently, a genetic predisposition to TAAs has been identified, highlighting the dominant role of gene mutations in the TGF-β signaling cascade and SMC contractile apparatus (Isselbacher et al., 2016). Additionally, research in experimental aneurysms has revealed hyperstimulation of the TGF-β pathway in TAAs (Neptune et al., 2003; Habashi et al., 2006), likely the result of genetic perturbations. Mutations of TGF-β signaling molecules are proposed to upregulate counter-regulatory pathways, such as those involving mitogen-activated protein kinase (MAPK), which can directly drive aneurysm development (Holm et al., 2011). Interestingly, TGF-β is poorly expressed in human AAAs and appears to play a protective role in their development. The modulation of TGF-β signaling prevents AAA formation in both elastase infusion and angiotensin II (AngII) perfusion animal models of AAA (Wang et al., 2010, 2013). On the other hand, mutations in the SMC contractile apparatus result in a loss of contractile structure, which is essential for the maintenance of an intact focal adhesion complex and the integrity of the ECM (Milewicz et al., 2008). It is currently unclear whether the relationship between such a contraction vasculopathy and TGF-β dysregulation correlates with - or is a precipitating cause of TAAs (Isselbacher et al., 2016), considering that TGF-β can both induce a phenotypic switch in SMCs and stimulate the secretion of matrix-degrading enzymes, such as matrix metalloproteinases (MMPs), from these cells(Renard et al., 2013).

## Abdominal aortic aneurysm

The defined mechanisms underlying AAAs are as well incompletely understood. AAA formation is thought to be a complex process, involving all vascular cell subtypes (Maegdefessel et al., 2013). The pathological features are characterized by endothelial dysfunction, chronic adventitial and medial inflammatory cell infiltration, ECM degradation, elastin fragmentation and medial devitalization, as well as SMC phenotype alterations (early stages) and apoptosis (late stages; Shimizu et al., 2006). Work in both common small animal models and humans has indicated that AAA development involves local inflammation and the infiltration of monocytes/macrophages, neutrophils, mast cells, and T and B lymphocytes (Sun et al., 2007). These infiltrating cells secrete various inflammatory cytokines and chemokines, such as interleukin (IL)-1β, IL-6, tumor necrosis factor (TNF)-α, and monocyte chemoattractant protein-1 (MCP-1), which can induce the activation of MMPs, particularly MMP-2 and MMP-9 (Golledge et al., 2006). These events contribute to ECM degradation and SMC depletion, leading to AAA progression and rupture.

## Non-coding RNAs

ncRNAs are RNA molecules that are not translated into protein products. They can be functionally classified into the following subgroups: gene expression regulation (miRNAs, piRNAs, lncRNAs), RNA maturation (snRNAs, snoRNAs), and protein synthesis (rRNAs, tRNAs; Fu, 2014). In this section, we will review some important aspects of miRNAs and lncRNAs, and highlight their regulatory roles in AA development and progression.

## MicroRNAs

MicroRNAs (miRNAs) are endogenous RNAs of ~22 nt that post-transcriptionally repress the expression of target genes, usually by binding to the 3′ untranslated region (UTR) of messenger RNA (mRNA). The stepwise progression of miRNA maturation has already been established (Lee et al., 2002, 2003). The first step is nuclear cleavage of primary-miRNA (pri-miRNA) by Drosha RNase III endonuclease, resulting in a 60–70-nt stem-loop intermediate known as miRNA precursor (pre-miRNA), which is then actively transported from the nucleus to the cytoplasm by guanosine triphosphate (GTP)-binding nuclear protein Ran (RAs-related Nuclear protein Ran-GTP) and the export receptor exportin-5. In the cytoplasm, pre-miRNA is further processed by the enzyme Dicer, producing an imperfect siRNA-like duplex that composes the mature miRNA. Fully processed miRNAs associate with, and serve as specificity determinants for, the Argonaute (Ago) family of proteins within the RNA-induced silencing complex (RISC; Hutvagner and Zamore, 2002). At sites with extensive pairing complementarity, miRNAs can direct Ago-catalyzed mRNA cleavage. More commonly, though, they direct translational repression, mRNA destabilization, or a combination of these two processes (Bartel, 2009; Djuranovic et al., 2011).

MiRNAs have emerged as key post-transcriptional regulators of gene expression in the past decade, playing important roles in various biological processes and in disease development and progression(Bartel, 2004; Ebert and Sharp, 2012; Mendell and Olson, 2012). For AAs in particular, several miRNAs have been identified as crucial regulators, orchestrating the functions of all subtypes of vascular cells (Table 1). Endothelial cell (EC) dysfunction and leukocyte infiltration, degradation of the ECM, and SMC depletion are regarded as three pathological hallmarks of AAAs (Guo et al., 2006). As the aneurysm miRNA signature may differ depending on disease localization and morphology (Busch et al., 2016), one should act with caution when elucidating and comparing deregulations examined in human aneurysms, animal models, or cultured cells *in vitro*.

**Table 1:** MicroRNAs involoved in aortic aneurysms.

**miRNAs**	**Type**	**Sample studied**	**Cellular origin**	**Regulation**	**Target genes**	**Related Functions**	**References**
miR-15a	AAA	Human whole aorta, HAoSMCs	VSMC	↑	CDKN2B	Promotes proliferation and decreases apoptosis of VSMC	Gao et al., 2017
miR-17 cluster	TAD	Human dilated aorta, HAoSMCs from BAV	VSMC	↑	TIMP1, TIMP2	Downregulates ECM	Wu et al., 2016
miR-21	AAA	Human and mouse whole aorta, HAoSMCs	VSMC	↑	PTEN	Promotes proliferation and decreases apoptosis of VSMC	Maegdefessel et al., 2012a
miR-24	AAA	Human and mouse whole aorta and plasma, HAoSMCs, macrophage	VSMC, macrophage	↓	CHI3L1	Inhibits vascular inflammation	Maegdefessel et al., 2014
miR-26a	AAA	Mouse whole aorta, HAoSMCs	VSMC	↓	SMAD1, SMAD4	Promotes proliferation and inhibits differentiation, apoptosis of VSMC, alters TGF-β signaling	Leeper et al., 2011
miR-29	AAA, TAA	Human and mouse whole aorta, HAoSMCs	Fibroblast, VSMC	↓	COL1A1, COL3A1, COL5A1, ELN, MMP2, MMP9	Downregulates ECM and reulates fibrosis	Boon et al., 2011; Maegdefessel et al., 2012b
miR-29a	TAD	Human aorta, HAoSMCs	VSMC	↓	MMP2, MMP9	Downregulates ECM	Jones et al., 2011
miR-29b	TAA	Fbn1(C1039G/+) aorta, HAoSMCs	VSMC	↑	ELN, MMP2	Upregulates ECM and promotes apoptosis of VSMC	Merk et al., 2012
miR-29c	AAA	Human serum, HUVEs	EC	↑	ELN, COL4A1, PTEN, VEGFA	Regulates ECM	Licholai et al., 2016
miR-98	AAA	THP-1, HAoSMCs	Macrophage, VSMC	-	-	MCP-1/miR-98/IL-6/p38 regulatory loop, induce VSMC apoptosis	Wang et al., 2015b
miR-129	AAA	Mouse whole aorta, HAoSMCs	VSMC	↓	Wnt5a	Inhibits proliferation and induce apoptosis of VSMC	Zhang et al., 2016
miR-143/145	TAD	Human TAD aorta, mouse aorta, HAoSMCs	VSMC	↓	Klf4, myocardin, Elk-1, SRF	Promotes differentiation and represses proliferation of VSMC	Lesauskaite et al., 2001; Elia et al., 2009; Liao et al., 2011
miR-155	AAA	Human and mouse whole aorta, human plasma, HAoSMCs	TC	↑ in tissue, ↓ in plama	CTLA4, SMAD2	Promotes vascular inflammation	Biros et al., 2014
miR-181b	AAA, TAA	Human and mouse whole aorta, HAoSMCs	Macrophage, VSMC	↑	TIMP3, ELN	Downregulates ECM	Di Gregoli et al., 2017
miR-195	AAA	Human and mouse whole aorta, HAoSMCs	VSMC	↑	COL1A1, COL1A2, COL3A1, FBN1, ELN, MMP2, MMP9	Regulates ECM	Zampetaki et al., 2014
miR-221/222	AAA	Human whole aorta, Rat SMCs and Ecs	VSMC, EC	↑	kip1, kip2, c-kit	Pro-proliferative, pro-migration, and anti-apoptotic effects, Promote a synthetic phenotype in VSMCs	Davis et al., 2009; Liu et al., 2012
miR-223	AAA	Human aorta and plasma, Rat Cerebral Aneurysms	Macrophage	↑ in tissue, ↓ in plama	MMP12	Inhibits vascular inflammation	Kanematsu et al., 2011; Kin et al., 2012; Lee et al., 2013
miR-516a	AAA	HAoSMCs	VSMC	↑	MTHFR, MMP2, TIMP1	Regulates ECM	Tung Chan et al., 2017
miR-712/205	AAA	Mouse whole aorta, HAoSMCs	EC, leukocytes	↑	TIMP3, RECK	Induces inflammation, regulate ECM	Kim et al., 2014

*AAA, abdominal aortic aneurysm; TAA, thoracic aortic aneurysm; TAD, thoracic aortic dissection; HAoSMCs, human aortic smooth muscle cells; HUVEs, human umbilical vein endothelial cells; VSMC, vascular smooth muscle cell, EC, endothelial cell; TC, T lymphocytes; COL1A1/2, collagen type 1 alpha 1/2; COL3A1/2, collagen type 3 alpha 1; FBN, fibrillin; ELN, elastin; kip1/CDKN1B, Cyclin-dependent kinase inhibitor 1B; kip2/CDKN1C, Cyclin-dependent kinase inhibitor 1B; c-kit/SCFR, Mast/stem cell growth factor receptor; ELK1, ETS domain-containing protein; KLF4, Kruppel-like factor 4; CTLA4, cytotoxic T-lymphocyte associated protein 4; MCL1, myeloid cell leukemia 1; MMP, matrix metalloproteinase; TIMP3, metalloproteinase inhibitor 3; PTEN, phosphatase and tensin homolog; CHI3L1, chitinase 3 Like 1; Wnt Wnt5a, Family Member 5A; SMAD, mothers against decapentaplegic homolog; RECK, reversion-inducing-cysteine-rich protein with kazal motifs; VEGFA, Vascular endothelial growth factor A; MTHFR, methylenetetrahydrofolate reductase. ECM, extracellular matrix; TGF-β, transforming growth factor-β*.

## MiRNAs, endothelial dysfunction, and vascular inflammation

ECs are the first cell sensors exposed to pathologic factors, such as hyperlipidemia and turbulent flow shear stress, in the lumen of the aorta. To address the role of endothelial miRNAs in AAA development, Kim et al. (2014) utilized the mouse AngII infusion model of AAAs, one of the most important experimental platforms to study the pathophysiology of AAAs. Using an miRNA microarray platform, the authors experimentally confirmed the upregulation of miR-712/205 in ECs as well as in the medial layer of the aorta. Of note, the two key inhibitors of MMPs, tissue inhibitor of metalloproteinase 3 (TIMP3) and reversion-inducing-cysteine-rich protein with kazal motifs (RECK), which lead to both the activation of MMPs in the aortic wall and the development of AAAs in the mouse AngII infusion model, were validated as direct targets of miR-712. In addition, the manipulation of both AngII and miR-712/205 affected the adhesion of circulating leukocytes, further highlighting the therapeutic potential of targeting this pathway, while implying that other mechanisms are involved. Furthermore, in human vascular ECs, other genes related to ECM synthesis and maintenance of ECM integrity, such as elastin (ELN), collagen type 4 alpha 1 (COL4A1), phosphatase and tensin homolo (PTEN), and vascular endothelial growth factor A (VEGFA), were recently identified as targets of miR-29c and are significantly elevated in the sera of AAA patients (Licholai et al., 2016).

Subsequent inflammatory cell infiltration may be facilitated by miR-155, whose levels were found to be highly increased in AAA biopsies and circulating sera in AAA patients (Biros et al., 2014). It is believed that miR-155 not only promotes chronic inflammation by enhancing T cell development via the downregulation of cytotoxic T-lymphocyte associated protein 4 (CTLA4), but also blocks mothers against decapentaplegic homolog (SMAD2) translation and protein synthesis during the regulation of TGF-β signaling, leading to AAA development. Similarly, miR-181b was shown to endow macrophages with more invasive and proliferative capabilities to control aneurysm progression via the negative regulation of tissue inhibitor of MMP-3 expression (TIMP3; Di Gregoli et al., 2017). Moreover, miR-223, a novel regulator of inflammation that inhibits proinflammatory pathways and enhances anti-inflammatory responses, was dysregulated in AAAs (Kin et al., 2012) and negatively correlated with MCP-1, TNF-α, and TGF-β expression in diseased aortic tissues(Kanematsu et al., 2011; Lee et al., 2013). Recently, miR-103a (Jiao et al., 2017) was reported as an “anti-aneurysmal” miRNA that suppresses inflammation within the aortic wall, limiting AAA formation and progression by downregulating the protease ADAM Metallopeptidase Domain 10 (ADAM10).

Our lab discovered miR-24 as a key regulator of vascular inflammation and AAA pathology (Maegdefessel et al., 2014). Before elucidating the highly conserved miR-23b-24-27b cluster in AAA diseases, others have shown that this miRNA family is involved in postinfarction cardiac angiogenesis (Fiedler et al., 2011), cardiomyocyte survival (Qian et al., 2011), cancer (Hatziapostolou et al., 2011; Qian et al., 2011), and atherosclerosis (Geng et al., 2016). The miR-23b-24-27b cluster has been further linked to inflammation by its ability to modulate the nuclear factor kappa-light-chain-enhancer of activated B cells (NF-κB) pathway in macrophages (Thulasingam et al., 2011). In addition, Hatziapostolou et al. (2011) demonstrated an miRNA-inflammatory feedback loop, consisting of miR-124, IL-6R, signal transducer and activator of transcription 3 (STAT3), miR-24, and miR-629, in which the systemic administration of miRNAs prevented and suppressed hepatocellular carcinogenesis by the induction of tumor-specific apoptosis without toxic side effects.

In our own study (Maegdefessel et al., 2014), we profiled miRNA expression and validated the downregulation of the miR-23b-24-27b cluster in murine AAA models, finding that miR-24 displayed the most significant inverse regulation with its predicted targets. Human AAAs also show evidence of miR-24 downregulation, which correlates inversely with aneurysm size. Further analysis suggested “chitinase 3-like 1′ (Chi3l1), a mediator/marker of inflammation, as an intriguing miR-24 target. Chi3l1 is secreted by differentiated macrophages in early stage atherosclerotic lesions and modulates SMC proliferation and migration (Rehli et al., 2003). In the context of AAAs, miR-24-Chi3l1 interactions appear to have broad effects on all cells present in the aortic wall, such as the regulation of macrophage survival and cytokine synthesis, promotion of aortic SMC migration and cytokine production, and stimulation of adhesion molecule expression in vascular ECs. Furthermore, overexpression of miR-24 in murine models was able to significantly decrease Chi3l1 levels, leading to arrested AAA development and reduced immune responses and cytokine activities, suggesting that miR-24 downregulation contributes to aneurysm growth. In contrast, the inhibition of miR-24 was shown to accelerate AAA progression by augmentation of the degree of inflammatory and apoptosis-related responses.

Consistent with findings related to miR-24 and the inflammation loop, Pua et al. (2016) provided evidence that miR-24 and miR-27 suppress allergic inflammation and target regulators of T helper 2 cell-associated cytokine production. These data suggest that miR-24 may be involved in leukocyte infiltration during aneurysm development. Another study from Geng et al. (2016) examined the pathological consequences of subclinical endotoxemia during both the low-grade inflammatory polarization of monocytes and the progression of atherosclerosis. These authors observed that subclinical endotoxemia was able to shift monocytes into a nonresolving inflammatory state, with elevated levels of lymphocyte antigen 6 complex locus C (Ly6C), chemokine (C-C motif) receptor 5 (CCR5), and MCP-1, and reduced levels of scavenger receptor class B member 1 (SR-B1), due to the disruption of homeostatic tolerance by elevated miR-24 levels and reduced levels of the key negative-feedback regulator interleukin-1 receptor-associated kinase (IRAK)-M. MiR-24 targets SMAD4, which is required for the expression of IRAK-M and the key lipid-processing molecule SR-B1. IRAK-M deficiency in turn leads to elevated miR-24 levels, sustaining the disruption of monocyte homeostasis and aggravating atherosclerosis. Another study recently confirmed the role of miR-24-Chi3l1 interactions in Staphylococcus aureus-stimulated macrophages (Jingjing et al., 2017). MiR-24 was found to decrease both M1 macrophage polarization and production of proinflammatory cytokines, and induce both M2 macrophage polarization and secretion of anti-inflammatory factors. These actions are mediated by the direct suppression of Chi3l1 and the inhibition of the MAPK pathway.

## MiRNAs and smooth muscle cell homeostasis

SMCs are the predominant cells in the tunica media of the aorta and are essential for the maintenance of aortic structure and function. The phenotype of SMCs can change from a contractile (differentiated) to a synthetic (dedifferentiated) state, and vice versa, in response to changing environmental conditions. The differentiated phenotype is characterized by high levels of contractile gene expression, with low rates of proliferation, migration, and ECM synthesis; the dedifferentiated phenotype has the opposite features. Dysregulation of both phenotype switching and SMC apoptosis contributes to the development and progression of AAs (Henderson et al., 1999; Ailawadi et al., 2009). Previous studies have revealed that SMC homeostasis is intensively regulated by a vast array of miRNAs (Table 1).

Leeper and collaborators (Leeper et al., 2011) were able to prove that miR-26a is an important regulator of the SMC phenotype. They performed a microarray-based study during human aortic SMC differentiation *in vitro* and showed that miR-26a was the highest-ranked significant miRNA present. SMC behavior was evaluated by the modulation of miR-26 via the transfection of either pre-miR or anti-miR. The decreased level of miR-26a was associated with a reduction in SMC proliferation and migration, and a significant increase in H_2_O_2_-induced apoptosis. Mechanistically, miR-26a targeted the expression of SMAD1 and SMAD4, members of the TGF-β signaling cascade, and thus affected AAA development. Moreover, the expression of miR-26a was found to be progressively downregulated in two murine AAA models, suggesting that miR-26a may represent a novel therapeutic target in conditions of pathologic aneurysmal dilation.

MiR-221/222, which are highly expressed in SMCs and ECs, seem to have a cell-specific effect on the aorta. Liu et al. (2012) isolated SMCs and ECs from the aortas of male Sprague-Dawley rats and assessed the cellular responses to miR-221/222. Interestingly, these miRNAs had pro-proliferative, pro-migratory, and anti-apoptotic effects on SMCs - but the completely opposite effect on ECs. MiR-221/222 appears to directly target and downregulate p27 (Kip1), p57 (Kip2), and c-Kit, three proteins that are involved in key processes of cell differentiation, proliferation, migration, and apoptosis, and are also differentially expressed in SMCs and ECs. Consistent with these data, Davis et al. (2009). identified that miR-221 was critical for the platelet-derived growth factor (PDGF)-mediated induction of cell proliferation by downregulating the expression of p27 (Kip1) and c-Kit.

In our previous work (Maegdefessel et al., 2012a), we discovered the essential role of miR-21 in SMC homeostasis during AAA development. MiR-21 is highly expressed in SMCs and is known to be involved in the regulation of SMC biology by targeting several cell fate-determination genes, such as PTEN (Horita et al., 2011), programmed cell death 4 (PDCD4; Liu et al., 2010), and B cell lymphoma 2 (BCL2; Ji et al., 2007). Ji et al. (2007) found that miR-21 was one of the most upregulated miRNAs in the vascular wall after balloon injury, and that miR-21 depletion significantly decreased neointima formation, suggesting that miR-21 serves as an important regulator of neointimal hyperplasia, which results from an imbalance between SMC proliferation and apoptosis. Indeed, these authors confirmed that PTEN and BCL2, two important signaling molecules associated with SMC growth and apoptosis, were miR-21 targets. In addition, evidence from the study of Davis et al. (2008) showed that miR-21 mediated the TGF-β- and bone morphogenetic protein (BMP)-induced contractile phenotype switch in human SMCs. Meanwhile, miR-21 downregulated PDCD4, which in turn acts as a negative-feedback regulator of smooth muscle contractile genes (Davis et al., 2008). These data together indicate that miR-21 regulates SMC contractile function, proliferation, and apoptosis.

We found that miR-21 expression was substantially increased as AAAs developed in both the porcine pancreatic elastase perfusion and AngII infusion AAA models. PTEN, a key negative regulator of the phosphoinositide 3-kinase pathway, was the only target significantly downregulated and was inversely correlated with miR-21 expression during AAA development and progression. Lentiviral overexpression of miR-21 limited AAA growth, which was associated with increased SMC proliferation and decreased levels of PTEN expression and apoptosis in the aortic wall. In contrast, systemic injections of a locked nucleic acid (LNA)-modified antagomir targeting miR-21 diminished the pro-proliferative impact of downregulated PTEN, leading to a marked increase in AAA size. Our *in vitro* studies also identified the transcription factor NF-κB as a crucial positive regulator of miR-21 expression in vascular cells. Nicotine, IL-6, and AngII were each able to induce miR-21 via the upregulation of NF-κB.

For translational purposes, the utilization of local delivery tools has been discussed previously and is under current investigation by our lab and others (Maegdefessel, 2014). Local delivery into the vasculature and diseased aorta appears feasible by using drug eluting stents and balloons, enabling miRNA-based therapies to avoid off-target effects in the respiratory, renal and cardiovascular system, as well as the liver and the spleen. For miR-21 inhibition, and its effects on SMC proliferation we have utilized an anti-miR-21-eluting stent to effectively prevent experimental in-stent restenosis without significant observable side effects (Wang et al., 2015a). However, to make balloon and/or stent- delivered miRNA modulators a real option for the future, additional obstacles have to taken, including optimal patient selection and timing of the intervention, as well as strategies how to handle disease-related barriers, such as intraluminal thrombi and the varying anatomy of different AAA. In relation to this, the development of novel (ideally patient-like) pre-clinical models of AAA, utilizing large animal models (e.g., landrace pigs and/or Yucatan mini-pigs), which based on their dimension and physiological properties (circulating blood volume, size of the aorta, blood pressure, etc.) are better suited for translational approaches to test novel therapies in aortic diseases.

## MiRNAs and extracellular matrix formation

The aortic wall is composed of three layers: the intima, the media, and the adventitia. Medial SMCs and an arrangement of ECM structural proteins, primarily collagen and elastin, are essential for the maintenance of the structure and function of the aorta. During AA development, the integrity of these layers and protein components are disrupted. Degradation of the ECM is one of the characteristics of AAs, yet the mechanisms underlying this process are incompletely understood. It has been suggested that TGF-β signaling plays a key role in ECM dysregulation and AAA development.

Among the miR-15 family members, miR-195 levels were shown to be significantly increased in the aortas of Apo E-deficient mice after AngII infusion. Consistent with the increased levels of miR-195 in dissected human aortas, the direct binding of miR-195 to several ECM transcripts was detected. Based on these findings, Zampetaki et al. (2014) conducted a proteomics analysis of the secretome of murine aortic SMCs after miR-195 manipulation and revealed that miR-195 targets a group of ECM proteins, including collagens, proteoglycans, elastin, and proteins associated with elastic microfibrils. The inhibition of miR-195 *in vivo* led to higher expression levels of aortic elastin, being related to increases in levels of both MMP-2 and MMP-9.

In human plasma, miR-195 expression was shown to lead to an inverse correlation between the presence of AAAs and aortic diameter (Zampetaki et al., 2014). These data indicated that miR-195 may contribute to the pathogenesis of AAAs via ECM dysregulation.

In addition to miR-195, previous data from our lab has illustrated a crucial role for miR-29b in ECM homeostasis and AAA development (Maegdefessel et al., 2012b). The miR-29 family of miRNAs (miR-29a, miR-29b, and miR-29c) have been reported to target various genes that encode ECM proteins involved in fibrotic responses, including several collagen isoforms (e.g., collagen types I and III), fibrillin-1, and elastin (van Rooij et al., 2006). A profibrotic response is usually considered a pathological feature, accompanied by significant malfunctions of affected organs, including the respiratory and renal system, as well as the heart and the liver. However, fibrotic responses and ECM deposition appear essentially beneficial for patients with AAs, based on the fact that one of the hallmarks of AAs is the constant degradation of the ECM.

Our data indicated that miR-29b was the only member of the miR-29 family whose levels were substantially decreased at three different time points during murine AAA development and progression. Anti-miR-29b treatment mainly increased the expression of genes encoding collagen (Col1a1, Col2a1, Col3a1, and Col5a1) and elastin, which accounted for limited aneurysm expansion. In contrast, the overexpression of miR-29b using a lentiviral vector led to rapid AAA expansion and an increased rate of aortic rupture. Furthermore, human AAA tissue samples displayed a similar pattern of reduced miR-29b expression with increased collagen gene expression in comparison to tissue samples from non-aneurysmal organ donor controls. Furthermore, cell culture studies identified aortic fibroblasts as the likely cell type mediating the profibrotic effects of miR-29b. Interestingly, TGF-β, a validated regulator of tissue fibrosis, can repress miR-29b expression, which suggests a protective role of TGF-β in limiting AAA development. A more detailed discussion of the miR-29 family in relation to aging and TAAs can be found below.

## MiRNAs in TAAs

The miR-143/145 cluster is highly expressed in SMCs; it is one of the most studied miRNAs in the regulation of SMC phenotype switching and vascular disease pathogenesis (Boettger et al., 2009). MiR-145 has been repeatedly shown to promote SMC differentiation and to inhibit SMC proliferation (Cheng et al., 2009; Cordes et al., 2009). Tunica media SMCs have been observed to transform from a contractile phenotype to a synthetic phenotype during thoracic aortic dissection (TAD; Lesauskaite et al., 2001). Elia et al. (2009) also found decreased miR-143 and miR-145 expression levels in human AAs, which subsequently induced structural changes in the aorta, due to the incomplete differentiation of SMCs. In addition, Liao et al. (2011) reported that miR-143 and miR-145 were downregulated in TAD, which may account for the underdifferentiation of SMCs and aortic remodeling.

Apart from the proinflammatory effects elicited by activated macrophages, miR-181b was also shown to be involved in ECM dysregulation during TAA development and progression. Prominent fragmentation of the elastic lamellae, a key feature of TAAs, was abrogated by the inhibition of miR-181b compared with control animals; increased TIMP3 expression and collagen accumulation were also observed (Di Gregoli et al., 2017).

As mentioned above, miR-29 family members have also been shown to participate in TAA development and progression. Boon et al. (2011) managed to link miRNA regulation to aortic dilatation and aging. The authors discovered that elevated expression levels of miR-29 family members were associated with a profound downregulation of numerous ECM components in the aortas of aged mice, as well as in two experimental models of aortic dilation (i.e., AngII perfusion and Fibulin4^R/R^ mice). Consistent with our results in AAAs (Maegdefessel et al., 2012b), LNA-modified antisense oligonucleotide-mediated silencing of miR-29 was found to induce collagen gene isoform expression and inhibit AngII-stimulated dilation of aortas in mice.

In a similar context, Merk and collaborators (Merk et al., 2012) further revealed that miR-29b participates in early aneurysm development in Marfan/Fbn1^C1039G/+^ mice. Blockade of miR-29b prevented early aneurysm development, cell apoptosis, and ECM deficiencies in the aortic root. These data suggest that miR-29 may represent a novel molecular target to augment matrix synthesis and maintain the structural integrity of the vascular wall.

A study from Jones et al. (2011) identified the role of miR-29a in TAA development. A significant inverse relationship between miR-29a and MMP-2 was identified in clinical TAA specimens, indicating that miR-29a may regulate ECM production by targeting MMP-2 during TAA development.

Recently, a new cluster of miRNAs (miR-17-associated miRNAs) was identified in progressive aortic dilation (Wu et al., 2016). Patients with a bicuspid aortic valve (BAV) are at increased risk for progressive aortic dilation associated with ECM degradation, relating to the increased activity of MMPs. In the heart, MMP activity is regulated via miR-17-enforced repression of TIMP, a mechanism the investigators were able to establish for the ascending aorta as well. MiRNA profiling of samples from dilated sections of ascending aortas indicated significantly increased miR-17 cluster expression levels in less dilated aortic segments compared to severely dilated segments. Both TIMP-1 and TIMP-2 were validated as miR-17 targets, and their expression levels were substantially decreased in the less dilated samples. As expected, the subsequent increased activity of MMPs was found to contribute to ECM disruption and aortic dilation. In addition, an miR-17 mimic decreased TIMP-1 and TIMP-2 expression and increased MMP2 activity in SMCs isolated from normal and BAV aortas, whereas the opposite effects were seen with an miR-17 inhibitor, suggesting that the miR-17-TIMP-MMP pathway mediates matrix degradation in progressive aortic dilation in BAV-associated AAs.

## Long non-coding RNAs

Long non-coding RNAs (lncRNAs) are generally defined as RNA transcripts with lengths >200 nt that do not encode proteins. They are involved in numerous biological events, such as signaling via scaffolding proteins, shaping nuclear architecture, imprinting genomic loci, and regulating enzymatic activity (Engreitz et al., 2016; Figure 1). The aberrant expression or mutation of lncRNA genes has been implicated in various human diseases (Batista and Chang, 2013). However, the functional roles and mechanisms of most lncRNAs remain elusive (Cech and Steitz, 2014).

Particularly in cardiovascular development and diseases, very few lncRNAs have been functionally characterized. Braveheart was the first lncRNA described in the heart, being required for cardiac development (Klattenhoff et al., 2013). Similarly, the lncRNA Fendrr regulates the transcriptional network required for cardiac development via interactions with histone-modifying complexes (polycomb Repressive Complex 2, PRC2 and trithorax group/myeloid/lymphoid or mixed-lineage Leukemia, TrxG/MLL) (Grote et al., 2013). There are other lncRNAs related to the differentiation of human embryonic stem cells, including TERMINATOR, ALIEN, and PUNISHER (Kurian et al., 2015).

During the development of cardiovascular diseases, lncRNAs seem to either serve as potential indicators (biomarkers) or regulators of cell function and disease progression. For instance, Novlnc6 was found to be differentially expressed in patients with dilated cardiomyopathy (Ounzain et al., 2015), and Hotair levels were reduced in patients with BAV and related valve calcifications (Carrion et al., 2014). The expression levels of the lncRNAs HIF1A antisense RNA (aHIF), antisense non-coding RNA in the INK4 Locus (ANRIL), KCNQ1 opposite strand/antisense transcript 1 (KCNQ1OT1), myocardial infarction associated transcrip (MIAT), and metastasis associated lung adenocarcinoma transcript 1 (MALAT1) may be associated with acute myocardial infarction(Vausort et al., 2014). MALAT1 regulates EC function and vascular inflammation in patients with diabetes-induced vascular complications (Michalik et al., 2014), while MIAT was identified as a competing endogenous RNA, that forms a feedback loop with VEGF and miR-150-5p and controls EC function in diabetic retinopathies (Yan et al., 2015). The long intergenic non-coding RNA (lincRNA)-p21 was shown to repress proliferation and induce apoptosis of SMCs in atherosclerotic plaques (Wu et al., 2014).

One particular lncRNA, H19, appears to have a universal effect on the cardiovascular system. The downregulation of H19 was shown to promote cell proliferation and inhibit cell apoptosis during late-stage cardiac differentiation by regulating the negative role of miR-19b in Sox6 expression (Han et al., 2016). H19 was also shown to mediate the inhibition of melatonin by inducing the premature senescence of c-kit+ cardiac progenitor cells via miR-675 stimulation (Cai et al., 2016). In addition, H19 inhibition was found to decrease human umbilical vein endothelial cells (HUVEC) growth and capillary formation, while the H19-miR-675 axis targets calcium/calmodulin dependent protein kinase II Delta (CaMKIIδ), thus serving as a negative regulator of cardiac hypertrophy (Liu et al., 2016). H19-derived miR-675 was found to aggravate restenosis by targeting PTEN in VSMCs (Lv et al., 2017). These data indicate that H19 participates in cardiac development and related cardiovascular disease processes. The altered DNA methylation of H19 in calcified aortic valve disease was shown to promote mineralization by silencing NOTCH1 (Hadji et al., 2016). LncRNAs that have been studied for their implications in SMC plasticity and AA development and dilation are summarized in Table 2.

**Table 2:** LncRNAs involoved in VSMCs and aortic aneurysms.

**LncRNAs**	**Sample studied**	**Regulation**	**Related genes**	**Related Functions**	**References**
ANRIL	human blood, mouse atherosclerotic plaque, HAoSMCs	↓	CDKN2A, CDKN2B, DAB2IP, LRP1, LRPR, CNTN3	Influences CDKN2A/B expression and promotes proliferation of VSMC	Congrains et al., 2012; Motterle et al., 2012
RNCR3	Human blood, mouse atherosclerotic plaque, HAoSMCs, ECs	↑	KLF-2, miR-185-5p	Acts as a ceRNAs, decreases EC and VSMC proliferation	Shan et al., 2016
H19	Balloon-injured artery, HAoSMCs	↑	miR-675	Generates miRNA, Promotes VSMC proliferation	Lv et al., 2017
Lnc-Ang362	mVSMCs	↑	miR-221/222	Produces miRs and promotes VSMC proliferation	Leung et al., 2016
SENCR	HAoSMCs	-	FLI1	Inhibits migration of VSMC	Bell et al., 2014
Lnc-GAS5	mouse ocular vessels, SHR rat artery, HUVECs, HAoSMCs	↓	β-Catenin	Regulates ECs activation and proliferation, VSMC phenotypic conversion, and EC-VSMC communication	Wang et al., 2016
Lnc-MEG3	HAoSMCs	-	p53, MMP-2	Promotes proliferation and migration and decrease apoptosis of VSMC	Liu et al., 2017
MYOSLID	HCASMC	-	MYOCD/serum response factor and TGF-β/SMAD	Regulates VSMC phenotype	Zhao et al., 2016
HIF1A-AS1	Human TAAs serum, HAoSMCs	-	BRG1, Casp3/8, BCL2	Inhibits proliferation and induced apoptosis of VSMC	He et al., 2015; Wang et al., 2015c

*ANRIL, antisense non-coding RNA in the INK4 locus; SENCR, smooth muscle and endothelial cell-enriched migration/differentiation-associated lncRNA; GAS5, long noncoding RNA–growth arrest–specific 5; Lnc-MEG3, long noncoding RNA–Maternally expressed gene 3; MYOSLID, MYOcardin-induced smooth muscle lncRNA; HIF1A-AS1, antisense hypoxia inducible factor 1 alpha anti sense RNA; mVSMC, mouse vascular smooth muscle cells; HAoSMCs, human aorta smooth muscle cells; HUVEs, human umbilical vein endothelial cells; HCASMC, human coronary smooth muscle cells; LRP1, low density lipoprotein receptor-related protein 1; LRPR, low density lipoprotein receptor; CDKN, cyclin-dependent kinase inhibitors; DAB2IP, DAB2 interacting protein; CNTN3, contactin-3; KLF-2, Kruppel-like factor 2; FLI1, friend leukemia integration 1 transcription factor; ceRNAs, competing endogenous RNAs; BCL2, B-cell lymphoma 2; BRG1, Brahma-related gene 1; TGF-β, transforming growth factor-β; SMAD, mothers against decapentaplegic homolog*.

## LncRNAs and smooth muscle cell fate

The lncRNA ANRIL was identified as a transcript within the chromosome 9p21 locus, in which genetic variants are believed to contribute to the risk of cardiovascular disease (Broadbent et al., 2008). The genomic region (9p21) also contains the cyclin-dependent kinase Inhibitor 2A (CDKN2A) and CDKN2B genes, which encode for the cell cycle regulators p16 (INK4a), p14 (ARF), and p15 (INK4b). Two independent studies by Congrains et al. (2012) and Motterle et al. (2012) demonstrated that the alleles linked to atherosclerosis-related phenotypes and risk of common carotid artery stenosis were consistently associated with lower expression levels of ANRIL. Knockdown of ANRIL in SMCs was shown to cause significant variations in the expression of CDKN2A/B and reduced cell growth, suggesting that ANRIL may have pro-proliferation effects on SMCs. Leeper and colleagues have shown that loss of CDKN2B is crucial for aneurysm development via promoting p53-dependent SMC apoptosis (Leeper et al., 2013). The role of ANRIL itself in AA development needs to be investigated further.

LncRNA-RNCR3 is another lncRNA that has been linked to atherosclerosis-related vascular dysfunction (Shan et al., 2016). RNCR3 expression is significantly upregulated in mouse and human aortic atherosclerotic lesions, and inhibition of RNCR3 was shown to be sufficient to accelerate the development of atherosclerosis, aggravate hypercholesterolemia and inflammatory factor releases, and decrease EC and VSMC proliferation. Mechanistically, RNCR3 acts as a competing endogenous RNA (ceRNA) and forms a feedback loop with Kruppel-like factor 2 and miR-185-5p to regulate cell function. This study illuminated one of the most common mechanisms by which lncRNAs can act as ceRNAs to decrease the targeting concentration of miRNA, ultimately resulting in the derepression of other mRNAs with common miRNA response elements (Tay et al., 2014).

Another proposed mechanism of action for lncRNAs describes how these molecules serve as hosts for miRNA transcription. Lv et al. (2017) found that lncRNA H19 and H19-derived miR-675 are overexpressed in the neointima of balloon-injured arteries. In principal, they were able to show that lncRNA H19 promoted the proliferation of SMCs in an miR-675/PTEN-dependent manner. Similar roles were detected for the lnc-Ang362, which is substantially increased in response to AngII treatment (Leung et al., 2016). Knockdown of lnc-Ang362 reduces the expression of miR-222 and miR-221, suggesting its function as a miRNA host transcript. In addition, a reduction in the transcript levels of lnc-Ang362 was associated with a decrease in cell proliferation, suggesting that it plays a role in cell growth. These data evoke tremendous interest, considering that one of the most popular murine models of AAA development is AngII infusion in Apo E^−/−^ mice.

Smooth muscle and endothelial cell enriched migration/differentiation-associated long non-coding RNA (SENCR) was identified from the RNA sequencing of human coronary artery SMCs (Bell et al., 2014). SENCR is transcribed in the cytoplasm from the 5′ end of the friend leukemia integration 1 (FLI1) gene and exists as two splice variants. Experimental knockdown of SENCR has been shown to result in the decreased expression of myocardin and numerous smooth muscle contractile genes, and the increased expression of a number of promigratory genes. Results from loss-of-function studies support the role of Sencr as an inhibitor of SMC migration. Whether this property of phenotype regulation plays a role in AA development awaits further investigation.

In terms of SMC phenotypic switching, lncRNA-growth arrest-specific 5 (GAS5) was reported to be involved in hypertension-related vascular remodeling via changes in endothelial activation and proliferation, SMC phenotype conversion, and EC-VSMC communication, primarily through β-catenin signaling (Wang et al., 2016).

Recently, Zhao et al. (2016) described a novel lncRNA, named myocardin-induced smooth muscle lncRNA (MYOSLID), which is related to SMC phenotype regulation. MYOSLID, a direct transcriptional target of both the MYOCD/serum response factor (SRF) and TGF-β/SMAD pathways, promotes SMC differentiation and inhibits SMC proliferation. Depletion of MYOSLID in SMCs was shown to not only disrupt actin stress fiber formation and block nuclear translocation of MYOCD-related transcription factor A (MKL1), but was also found to abrogate TGF-β1-induced SMAD2 phosphorylation. These data suggest that MYOSLID may play a role in AA development via the crosstalk between the dysregulation of the VSMC phenotype and TGF-β signaling.

## LncRNAs and AAs

HIF1 alpha-antisense RNA 1 (HIF1A-AS1) was the first reported lncRNA that was found to play a role in TAA pathogenesis (Wang et al., 2015c). The expression of HIF1A-AS1 was shown to be regulated by Brahma-related gene 1, whose levels are elevated in TAAs. Suppression of HIF1A-AS1 was found to result in reduced apoptosis and increased proliferation of VSMCs. Furthermore, the expression of HIF1a-AS1 was reported to be significantly increased in sera from TAA patients (He et al., 2015). Knockdown of HIF1A-AS1 was shown to decrease the expression of caspase-3 and caspase-8, increase the expression of BCL2, and attenuate palmitic acid (PA)-induced cell apoptosis in SMCs. Loeys-Dietz syndrome (LDS) is an autosomal dominant genetic connective tissue disorder, and most LDS patients will commonly develop an aneurysm in the aortic root. Yu et al. (2015) found that the lncRNA AK056155 was up-regulated in LDS specimen, which correlated with an activation of the AKT/PI3K and TGF-β1 signaling.

Currently, there are no available publications of the roles of lncRNAs in AAAs. One paper from Falak et al. (2014) suggested that a putative lincRNA within the linkage region of protease inhibitor 15 (Pi15), a candidate gene marker for the risk of abdominal aortic internal elastic laminal ruptures in rats, may be involved in the regulation of Pi15 expression, providing some clues about the roles of lncRNAs in the initiation of human AAAs.

## Summary and perspective

**Figure 2:**
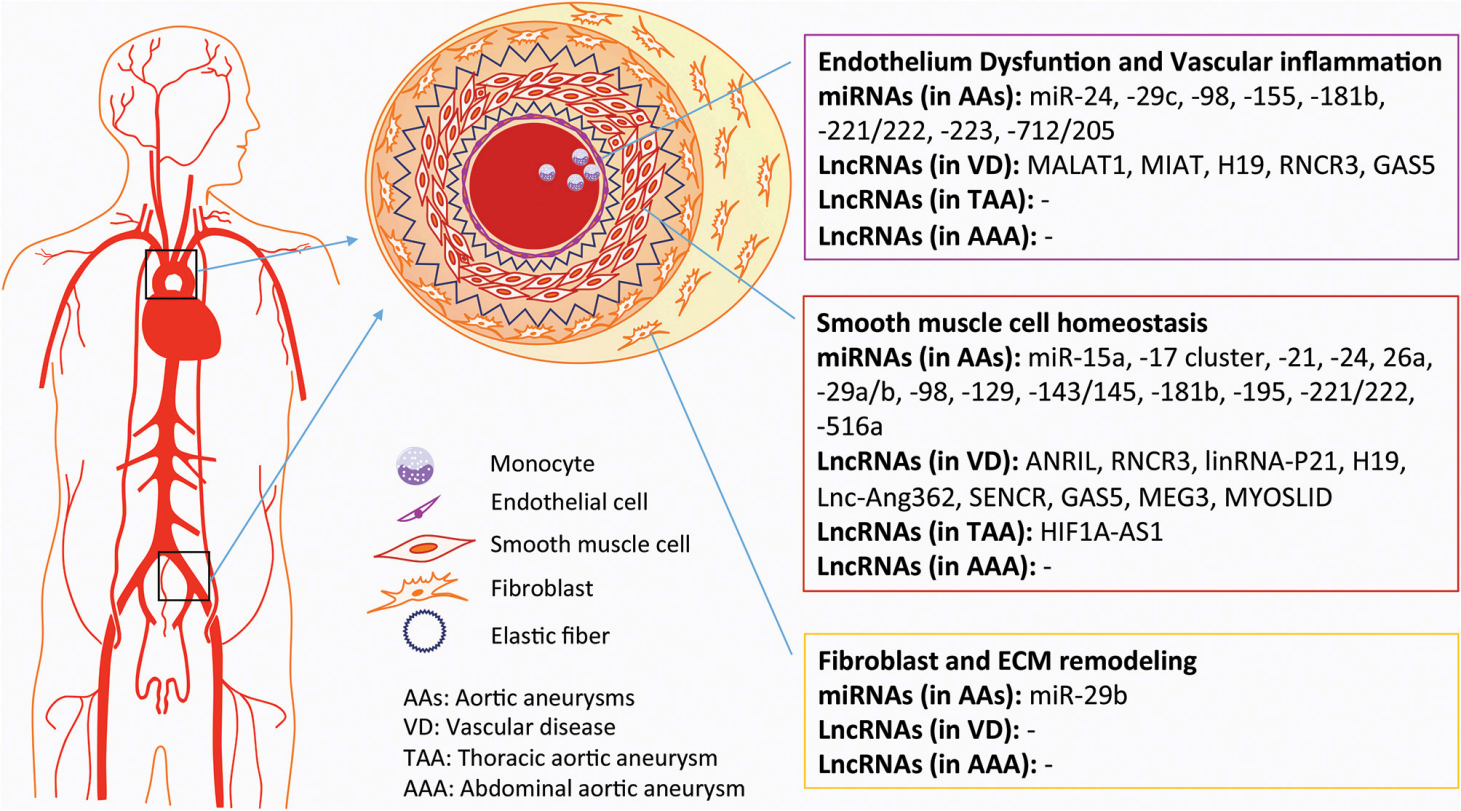
Non-coding RNAs involved in vascular disease and aortic aneurysms.

Today, undiscovered asymptomatic AAs are still considered to be ticking time bombs, owing to their incredibly high rates of mortality (~80%) when ruptured. Current clinical management of AAs, based only on size, is not satisfactory. Thus, extraordinary efforts have been launched to determine the pathophysiological characteristics and molecular events of the diseased aorta. Increasing evidence suggests that ncRNAs play crucial roles throughout AA development and might serve as therapeutic targets (Figure 2). As mentioned above, the pathogenesis and molecular mechanisms of TAAs and AAAs are distinguishable, suggesting that the roles of ncRNAs will be as well. As the field of miRNAs gradually matures, some promising candidates, such as miR-122, miR-21, miR-15, and miR-34 (Janssen et al., 2013; Christopher et al., 2016), may be further investigated in preclinical and clinical trials. The combined modulation of miRNAs in specific cell subtypes, like increasing SMC survival by miR-21 and inducing adventitial fibrosis by inbibiting miR-29b, would be worthwhile investments in AAA therapy.

The field of lncRNAs is still in its infancy and faces many challenges. First, the conservation of lncRNAs among species is not as high as that of miRNAs, and humanized models or organoid cultures will be necessary to study primate-specific lncRNAs. Second, mechanisms of action of lncRNAs are complicated and may be organ- or cell-specific. Third, the molecular mechanisms may be broadly diverse, based on the subcellular localization of lncRNAs and the existence of disparate transcriptional variants. Single-cell profiling and single-molecule imaging techniques are crucial to the detailed identification of components of these processes and machineries. Finally, a more efficient and specific delivery system is required for the modulation of lncRNAs *in vivo* to minimize off-targets and translate findings into clinical practice.

## Author contributions

Both authors listed contributed to this manuscript by conceptually designing and drafting the manuscript. Both authors have seen and approved the article for submission to Frontiers in Physiology.

### Conflict of interest statement

The authors declare that the research was conducted in the absence of any commercial or financial relationships that could be construed as a potential conflict of interest.
